# Blood Lactate AUC Is a Sensitive Test for Evaluating the Effect of Exercise Training on Functional Work Capacity in Patients with Chronic Heart Failure

**DOI:** 10.1155/2021/6619747

**Published:** 2021-09-30

**Authors:** Torstein Valborgland, Kjetil Isaksen, Peter Scott Munk, Alf Inge Larsen

**Affiliations:** ^1^Department of Cardiology, Stavanger University Hospital, Stavanger, Norway; ^2^Institute of Clinical Science, University of Bergen, Stavanger, Norway; ^3^Hospital of Southern Norway, Kristiansand, Kristiansand, Norway

## Abstract

**Purpose:**

Exercise training is an essential treatment option for patients with chronic heart failure (CHF). However, it remains controversial, which surrogate measures of functional work capacity are most reliable. The purpose of this paper was to compare functional capacity work measured as capillary lactate concentrations area under the curve (AUC) with standard cardiopulmonary exercise testing (CPET) with VO_2peak_ and the 6-minute walk test (6 MWT).

**Methods:**

Twenty-three patients in New York Heart Association (NYHA) class II/III with left ventricular ejection fraction (LVEF) <35% were randomised to home-based recommendation of regular exercise (RRE) (controls), moderate continuous training (MCT) or aerobic interval training (AIT). The MCT and AIT groups underwent 12 weeks of supervised exercise training. Exercise testing was performed as standard CPET treadmill test with analysis of VO_2peak_, the 6 MWT and a novel 30-minute submaximal treadmill test with capillary lactate AUC.

**Results:**

All patients had statistically significant improvements in VO_2peak_, 6 MWT and lactate AUC after 12 weeks of exercise training: 6 MWT (p =0.035), VO_2peak_ (p =0.049) and lactate AUC (p =0.002). Lactate AUC (p =0.046) and 6MWT (p =0.035), but not VO_2peak_ revealed difference between the exercise modalities regarding functional work capacity.

**Conclusion:**

6-MWT and lactate AUC, but not VO_2peak_, were able to reveal a statistically significant improvement in functional capacity between different exercise modalities.

## 1. Introduction

Maximal oxygen consumption (VO2_peak_) obtained during a cardiopulmonary exercise test (CPET) is considered an important prognostic indicator for morbidity and mortality [1, 2] in patients with chronic heart failure (CHF), and it is regarded the gold standard for assessing functional work capacity (FWC). The six minute walk test (6 MWT), which is easily available and highly reproducible, has also been shown to be a valuable prognostic marker comparable to VO2_peak._ [3]. Furthermore, this test has been demonstrated to be more sensitive to detect clinical meaningful changes in functional capacity after exercise interventions in the CHF population [4] compared to VO2_peak_, [5].

*Capillary blood lactate measurements* are considered superior to VO2_peak_ when evaluating endurance performance in homogenous groups of healthy athletes [6]. In line with this, calculated lactate area under the curve (AUC), using serial capillary blood lactate samples, is also a sensitive marker for changes in FWC after exercise intervention in CHF patients [7]. However, rather than measuring the *lactate threshold* as usually done in healthy subjects, the current test protocol measures *serial blood lactate levels* during a fixed submaximal exercise performance. The lactate AUC results from baseline during a 30 min submaximal treadmill test is compared with the lactate AUC following an exercise intervention program. There are no additional reports on this test, and the use of the method is not documented in any randomised trials among patients with CHF on modern optimal medical therapy (OMT). Therefore, the aim of the current study was to compare the sensitivity of 3 tests assessing FWC after a 3-month ET programme: blood lactate AUC, CPET (VO2_peak_) and 6 MWT. In particular, we wanted to assess if blood lactate AUC might be a sensitive tool in detecting effects of different exercise training modalities on FWC in this population.

## 2. Methods

### 2.1. Patients

In this prospective exercise training (ET) pilot study we randomised 23 patients with left ventricle ejection fraction (LVEF) <35% to either recommendation of regular exercise (RRE), moderate continuous training (MCT) or aerobic interval training (AIT). Because ET has a class 1A recommendation in current guidelines, controls could not be advised to inactivity, but rather to RRE. Randomization was 1 : 1 : 1 in three groups, where only MCT and AIT participated in a structured heart rate monitored and supervised exercise training programme. For baseline characteristics see Table 1. This was a sub-study of the SmartEx trial (Clinical trials.gov NCT00917046) conducted to test if AIT would be superior to MCT in reversing cardiac remodelling measured as a reduction in left ventricular end diastolic diameter (LVEDD) and improving VO2_peak._ [8]. The randomization and inclusion criteria have been described elsewhere [8–11]. Briefly, participants were selected based on symptoms of heart failure equivalent to NYHA II or III and reduced ejection fraction (HFrEF). All patients had been on optimal medical therapy for at least 12 weeks. Any planned revascularization, device implantation or recent surgery were exclusion criteria. In addition, participants with clinical unstable conditions, whether suffering from serious arrhythmia, significant valve disease, peripheral vascular disease or any other significant illness that would affect training capabilities were excluded. One participant was lost to follow up for the lactate test because of extreme worsening of heart failure and another patient refused to perform lactate test at 12 weeks. All patients signed informed consent form and the study was in accordance with the ethical standards of the institutional and national research committee, and with the 1964 Helsinki declaration and its later amendments or comparable ethical standards.

#### 2.1.1. Cardiopulmonary Exercise Testing

All tests were performed at baseline and after three months of exercise training. CPET was performed on a Star Track 3028 treadmill using 20 W/ramp protocol. Gas exchange data were collected continuously with an automated breath-by-breath system (System 2001; Medical Graphics Corporation, St Paul, MN, USA). VO2_peak_ and maximal heart rate was determined as the mean of the three highest 10-second measurements. Maximal workload was calculated by inclination degree and speed.

#### 2.1.2. Blood Lactate Area under the Curve

This test was performed on the same treadmill as used for the CPET. Patients exercised for 30 minutes at approximately 80% of maximal heart rate (based on baseline CPET data) throughout the test [7]. The baseline and follow-up test after 3 months of ET was performed at the exact same speed and treadmill inclination, intending equal resistance before and after the intervention period. Capillary blood from the tip of the index finger was drawn to measure lactate concentrations at rest, prior to exercise, every 4th minute during the test and 5 minutes after exercise testing. All capillary blood tests were analysed by Accutrend Plus (Roche Diagnostics). All lactate values of each patient were plotted for every 4^th^ minute during the test and the area under the curve was calculated. The area under the lactate curve was approximated by connection the measurement points by straight lines and then calculating the area under the resulting curve.

#### 2.1.3. 6-Minute Walk Test

The test was carried out on a 25-meter straight stretch before turning. Patients were given standardized instructions. “You will **walk** along this hallway between the marks as many times as you can in **6 minutes**. I will let you know as each **minute** goes past, and then at **6 minutes** I will ask you to stop where you are”. The same investigator performed all tests to obtain uniform instructions.

#### 2.1.4. Exercise Training (Training Protocols)

Experienced physiotherapists at the Outpatient Cardiac Rehabilitation Centre at Stavanger University Hospital led the monitored exercise intervention. The patients were randomised into three groups, where RRE patients were advised to perform ET at home. Subjects in the MCT and AIT groups participated in a structured and heart rate monitored ET programme three times a week lasting for 12 weeks. Exercise sessions were conducted on treadmills with a 1 to 1 monitor set up; data from the baseline CPET test were used to determine ET intensity (inclination and speed) in the beginning and subsequently adjusted to ensure that every training session was carried out at the correct intensity level. All subjects used a heart rate monitor to assess the predefined exercise intensity. To briefly summarize the key concept, AIT was performed by four 4-minute intervals at 90% – 95% of peak heart rate (HR_peak_) combined with 3-minute active recovery periods of moderate intensity, comprising a 38-minute session including warm-up and cool down. MCT exercised at 60% – 70% of HR_peak_ for 47 minutes. RRE patients were advised and motivated to exercise at home according to current recommendations and attended a session of moderate intensity training at 50% – 70%of peak heart rate every 3 weeks. A nurse checked patients in the AIT and MCT group briefly for adverse events before training sessions every 2 weeks. For practical reasons, RRE patients were checked at the four scheduled training sessions.

#### 2.1.5. Echocardiography

Subjects were examined in the left lateral supine position using the Vingmed Vivid 9 system (GE Vingmed Ultrasound, Horten, Norway). The following modes were obtained: parasternal long and short axis as well as M-mode. Apical 4-chamber, 2 chamber and apical long axis views were acquired, and left ventricle volumes calculated by Simpson biplane method. Left ventricle end-diastolic diameter (LVEDD) was measured at the tip of the mitral leaflet in parasternal long axis view.

#### 2.1.6. Statistical Analysis

Paired student t tests were used to analyse all patient from baseline to 12 weeks follow-up and to compare the two most active training groups (MCT and AIT) to the home-based training group (RRE).

All three groups were analysed with mixed two ways ANOVA to detect changes between time points and groups. As this was a sub study of the larger SmartEx trial and considered a pilot study, power analysis for changes in lactate AUC was not carried out.

Data analyses were performed with IBM SPSS Statistics for Macintosh version 25 (Armonk, NY: IBM Corp. Released 2017). Data were expressed as mean with standard deviation (SD) unless otherwise specified. All tests were two-tailed and a p-value <0.05 level was considered significant.

## 3. Results

All 23 patients completed the exercise-training programme with over 80% attendance to training sessions.

As depicted in Table 2, regardless of training modality, after 12 weeks of ET there was a statistically significant difference in all measures of FWC (6 MWT, VO2 _peak_ and blood lactate AUC), signifying improved aerobic fitness.

An ANOVA analysis (Table 3) comparing test modalities in the three groups confirmed a statistically significant difference with respect to the 6 MWT F (2, 20) =3.995, p =0.035, and for blood lactate AUC F (2, 20) = 3,678, p =0.046. No significant effect was seen with the VO2_peak_ analysis. Employing pairwise comparisons to detect a statistically significant effect of difference between groups showed that there was a difference in delta 6 MWT between RRE and the two training groups and also between MCT and the two other groups.

## 4. Discussion

The novel findings in this study were that blood lactate AUC during a submaximal exercise test was a sensitive marker for changes in FWC. Blood lactate AUC was at least comparable with standard tests such as 6 MWT and VO2_peak_ to detect effect after an ET programme. Further, blood lactate AUC and 6 MWT were the only tests that detected a significant difference in FWC between training modalities.

### 4.1. VO2_peak_ Test Vs 6 MWT

Despite a 1 A treatment recommendation for exercise training in both the European and American heart failure guidelines [12, 13] both training modalities and test protocols are still under discussion. In a sub study of the large HF-Action trial, Flynn et al. showed that a change in VO2_peak_ of only 0,26 mL/kg/min corresponds to 90 m change in 6 MWT, which in turn correspond to a 5-point change in Kansas City Cardiomyopathy Questionnaire (KCCQ). The authors therefore claimed that the KCCQ test and 6 MWT, in contrast to VO2_peak_, are clinical useful and meaningful tests for assessing effect on FWC in CHF, [5]. Despite the prognostic importance for both morbidity and mortality of the VO2_peak_ test, it has a considerable within subject variability when serial testing is done, as confirmed in another sub study of HF Action, in which the within subject variability was calculated to be 1.3 mL/kg/min with 48% to increase and 46% to decrease on the second test [14]. This variation weakens the tests ability to detect small changes in cohorts with few participants and might partly explain the neutral VO2_peak_ findings with regard to differences between the different ET modalities in the current study (Table 3).

In a third publication from the HF-Action trial, the researchers documented a strong predictive power of 6 MWT for both morbidity and mortality; comparable to the predictive power previously shown for VO2_peak_ [3]. This was also confirmed by Ingle et al., who showed that 6 MWT was an independent predictor of mortality in heart failure patients [15]. In addition, correlation tests in health related quality of life as a function of underlying physiological status shows only a modest correlation between self-reporting KCCQ and VO2_peak_ and 6 MWT with the highest correlation for 6 MWT [16]. This indicates that changes in VO2_peak_ may be clinically less important compared to changes in 6 MWT. However, despite several large trials there is still no consensus regarding what test is best in quantifying exercise intolerance in CHF patients. The findings of a statistically significant difference in *6 MWT* between groups employing both pairwise and group wise comparisons in the current study are in accordance with these recent publications underlining the sensitivity of this simple test.

### 4.2. Blood Lactate AUC as a Measure of Functional Work Capacity in Chronic Heart Failure

In patients with CHF there is a significant rise in serum lactate even during light to moderate exercise [17–19]. This increase is a continuum rather than a threshold, since anaerobic and aerobic pathways does not change suddenly during submaximal efforts [6, 20]. Serial measures of blood lactate during aerobic exercise of increasing intensity, is an established method to evaluate effects of exercise training among healthy athletes. Such testing is referred to as lactic acid threshold and the test modality measures the intensity level at which an exponential rise in blood lactate occurs in response to an increase in exercise intensity. Our test is performed at moderate continuous intensity and it is therefore not a threshold test. We use the exact same resistance at baseline as in the follow up test. The attenuation in measured blood lactate AUC will therefore correspond to the effect of the ET intervention. There is only one previous report on the use of this test modality, measuring steady state blood lactate AUC during moderate intensity aerobic exercise, to assess FWC in CHF [21].

**Figure 1:**
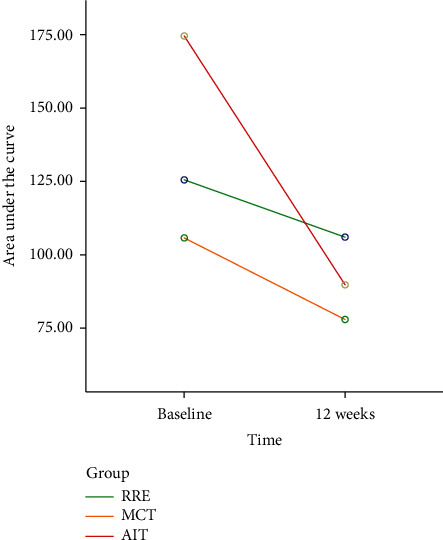
Area under the curve is expressed as Lactate in (mmol/L) x minutes and plotted at baseline and 12 weeks on the *x*-axis. RRE is recommendation of regular exercise training home-based exercise training without supervision. MCT is moderate continuous exercise training. AIT is aerobic interval training.

In the current study we observed a statistically significant drop in s-lactate AUC following an exercise intervention program, matching the significant increase in the 6-MWT. In addition, to being very sensitive, the lactate AUC test is not depending on a strenuous maximal exertion as is the case with a CPET examination. Moreover, it is relatively cheap and simple to perform compared with the high cost and complex set up of a CPET VO2_peak_ test. Additionally, the test with its steady state submaximal effort is less dependent on patient motivation when compared to VO2_peak_ test, but even more so with regard to the 6 MWT where one does not know by objective means if the patient is fully exhausted. In contrast to lactic acid threshold testing, blood lactate AUC is easier to perform, and as is the case with both the 6 MWT and CPET, it is also less strenuous. From a physiological point of view both tests compare blood lactic acid at, respectively, increasing and fixed intensities to evaluate adaptations to exercise. In the case of athletes increased fitness is reflected through an increased lactic acid threshold, meaning that a greater workload is required to trigger the exponential lactic acid increase. Among CHF patients we detect reduced levels of blood lactate calculated as AUC. As is known from exercise physiology with regard to improvements in lactic acid threshold, we detected the biggest decrease in lactate AUC among patients who exercised by the high intensity AIT exercise modality in the current study. We noted this difference despite no significant differences in VO2_peak_ detected between the exercise modalities. Several ET studies on CHF and patients with ischemic heart disease comparing MCT and AIT by means of VO2_peak_ have reached this same result [8, 22]. In fact, Figure 1 illustrates that the AIT group had a substantial 49% fall in blood lactate AUC compared to the MCT group 27%. Historic data from the ET intervention performed in 2001 employing MCT showed a 20% reduction in lactate AUC. This might indicate that AIT may be particularly effective in inducing lactate level attenuations in the CHF population, as shown in healthy individuals [23].

## 5. Conclusion

In this pilot trial measures of blood lactate calculated as AUC during an endurance treadmill submaximal effort was a sensitive and simple to perform test. The test was able to detect differences in FWC between exercise modalities not detectable with VO2_peak_ after an exercise training intervention in patients with CHF on OMT. The blood lactate AUC test might be particularly suitable to reveal small but clinically significant effects after ET interventions in the CHF population.

## 6. Limitations

The main limitation of this study is the low number of participants reducing its statistical power. An additional limitation is that the nature of the exercise intervention does not allow blinding of the randomised groups.

## Figures and Tables

**Table 1:** Baseline characteristics.

	Recommended regular exercise *n* = 8	Moderate continuous training *n* = 9	Aerobic interval training *n* = 6
Male/female	8/0	8/1	6/0
Age, mean (±SD)	65.1 ± 10.8	64.1 ± 5.2	66 ± 11.6
BMI, mean (±SD)	26.5 ± 3.4	26.8 ± 2.1	28.1 ± 5.1
*Heart failure parameters*			
IHD/DCM	6/2	6/3	4/2
NYHA class II/III	5/3	9/0	4/2
LV EF, % (±SD)	25.8 ± 5.2	26.0 ± 4.9	27,8 ± 5.4
LV EDD, mm (±SD)	63 ± 5	59 ± 7.2	64 ± 13.6
ICD/CRT	1/3	1/2	0/2
*Comorbidities*			
Smoking ex/current	2/1	4/1	3/3
COPD	0	0	1
Diabetes type I/II	1/0	0/0	0/2
History of hypertension	1	4	2
AF, paroxysmal/persistent	2/0	3/2	1/1
*Medication*			
Beta blockers (%)	8 (100.0)	8 (88.9)	5 (83.3)
ACE inhibitor (%)	5 (62.5)	3 (33.3)	5 (83.3)
ARB (%)	3 (37.5)	6 (66.7)	1 (16.7)
Aldosterone antagonist (%)	3 (37.5)	7 (77.8)	5 (83.3)
Diuretics (%)	3 (37.5)	6 (66.7)	4 (66.7)
Acetylsalicylic acid (%)	6 (75.0)	6 (66.7)	4 (66.7)
Clopidogrel (%)	0 (0.0)	1 (11.1)	2 (33.3)
Warfarin (%)	2 (25.0)	3 (33.3)	1 (16.7)
Statins (%)	7 (87.5)	7 (77.8)	5 (83.3)

No statistically significant differences between groups in any baseline characteristic. Numbers are frequencies unless otherwise stated. SD: standard deviation, BMI: body mass index, IHD: ischemic heart disease, DCM: dilated cardiomyopathy, NYHA: New York Heart Association, LV: left ventricular, EF: ejection fraction, EDD: end diastolic diameter, ICD: Implantable cardioverter defibrillator, CRT: cardiac resynchronisation therapy, COPD: chronic obstructive pulmonary disease, AF: atrial fibrillation, ARB: angiotensin receptor blocker.

**Table 2:** Difference from baseline to 12 weeks, all exercise modalities pooled.

	Baseline	12 weeks follow up	P
VO2_peak_ (n = 22) (ml/kg/min)	18.8 ± 4.0	19.6 ± 4.8	0.049∗
6 MWT (n = 21) (meters)	514 ± 66	545 ± 106	0.035∗
Lactate AUC(n = 21) (mmL x minutes)	132 ± 60	91 ± 25	0.002∗

Data are presented as mean ± standard deviation. ∗P- values calculated by paired student-t test for the three different test modalities. VO2_peak_: peak oxygen uptake. Lactate AUC: Lactate under the curve. 6 MWT: 6-minute walk test.

**Table 3:** Comparisons of training effect in two supervised intervention groups and controls.

	Recommended regular exercise n = 8		Moderate continuous training n = 9		Aerobic interval training n = 6	
	Baseline	12 weeks	Baseline	12 weeks	Baseline	12 weeks
VO2_peak_ (ml/kg/min)	17.0 ± 5.2	17.9 ± 6.4	21.3 ± 1.2	22.7 ± 2.0	17.6 ± 3.6	17.2 ± 2.8
6 MWT (meters)	492 ± 56	476 ± 115∗	554 ± 75	609 ± 89∗	483 ± 33	542 ± 61∗
Lactate AUC (mmol/L x minutes)	126 ± 43	106 ± 24∗	107 ± 36	78 ± 20∗	175 ± 84	90 ± 25∗

Mixed ANOVA is used to detect differences between groups. Data are mean ± SD. VO2_peak_ is maximum oxygen uptake. Lactate AUC is Lactate area under the curve. ∗Difference from baseline between groups p < 0.05.

## Data Availability

Data include sensitive and personal information and will not be shared as supplemental or underlying data.
